# High incidence of late effects found in Hodgkin's lymphoma survivors, following recall for breast cancer screening

**DOI:** 10.1038/sj.bjc.6602974

**Published:** 2006-02-07

**Authors:** D M Greenfield, J Wright, J E Brown, B W Hancock, H A Davies, L O'toole, C Eiser, R E Coleman, R J Ross

**Affiliations:** 1 Academic Unit of Clinical Oncology, Cancer Research Centre, University of Sheffield, Weston Park Hospital, Whitham Road, Sheffield S10 2SJ, UK; 2 Sheffield Teaching Hospitals NHS Foundation Trust, Royal Hallamshire Hospital, Glossop Road, Sheffield S10 2JF, UK; 3 Sheffield Children's Hospital Trust, Western Bank, Sheffield S10 2TH, UK; 4 Department of Psychology, University of Sheffield, Western Bank, Sheffield S10 2TN, UK; 5 Division of Clinical Sciences (North), University of Sheffield, Northern General Hospital, Herries Road, Sheffield S5 7AU, UK

**Keywords:** breast cancer, Hodgkin's lymphoma, late effects, mantle radiotherapy

## Abstract

Assessment of late effects in a cohort of female Hodgkin's lymphoma patients treated with mantle radiotherapy, identified from the DoH breast cancer screening recall showed high mortality and frequent undiagnosed abnormalities in tissues affected by radiotherapy. With increasing age, this patient group may suffer premature cardiac and respiratory morbidity.

Prolonged disease-free survival is attainable in up to 90% of patients presenting with early-stage Hodgkin's lymphoma (HL) (Henry-Amar and Joly, 1996). Current treatment usually involves multiagent chemotherapy combined with limited field radiotherapy in selected patients. However, until recently, the standard radiotherapy regimen for supradiaphragmatic HL was the mantle field (Deniz *et al*, 2003; Horwich and Swerdlow, 2004).

Young women who received mantle radiotherapy are at increased risk of breast cancer (Swerdlow *et al*, 2000; Deniz *et al*, 2003; Travis *et al*, 2003; Horwich and Swerdlow, 2004; Kenney *et al*, 2004). The risk is proportional to radiation dose, and time from treatment (Hancock *et al*, 1993a; Wolden *et al*, 1998). Younger age at the time of treatment gives the greatest risk (Hancock *et al*, 1993a; Bhatia *et al*, 1996). At 25 years of follow-up, the cumulative risk of breast cancer for women treated between 10 and 19 years of age is reported as 15–33%, and for those treated between the ages 20 and 29 years, 15–25% (Horwich and Swerdlow, 2004). In 2003, the UK Department of Health launched a ‘Patient Notification Exercise’ to inform patients of the increased breast cancer risk. The exercise mandated recall of all women with HL, who were diagnosed at or below the age of 35 from 1962 onwards. Mantle radiotherapy is associated with other long-term complications including second malignancies, and disorders of the thyroid, heart and lung (Morgan *et al*, 1985; Gustavsson *et al*, 1990; Allavena *et al*, 1992; Gustavsson *et al*, 1992; Henry-Amar and Joly, 1996). The aim of our study was to investigate the incidence of late effects in women recalled in the UK ‘Patient Notification Exercise’.

## MATERIALS AND METHODS

Patients were identified from a network database, according to the following criteria: diagnosis of HL after 1962 and before the age of 35 years, and mantle radiotherapy. Women with a known history of breast cancer were excluded. Patients were offered a clinic appointment for counselling regarding breast cancer risk. Patients who accepted were asked if they were willing to participate in a study of late effects. The study was approved by the South Sheffield Research Ethics Committee and all patients gave written informed consent.

### Late effects study protocol

Patients underwent a clinical evaluation including examination of the skin and thyroid. In addition, the following investigations were performed:

*Blood*: Serum thyroid-stimulating hormone (TSH), free T4 (fT4), parathyroid hormone and corrected calcium.
*Cardiac*: Echocardiogram and electrocardiogram.
*Respiratory*: Spirometry and diffusion capacity (TLCO).

### Measurement and interpretation of results

Serum measurements were considered against the laboratory normal range. Spirometry and transfer factors results were expressed as the percentage of predicted. All cardiology and respiratory tests were reviewed by a single cardiologist and respiratory physiologist, respectively. Tests were reviewed without the knowledge of patients' characteristics or other study data.

Cardiac and respiratory abnormalities were classified as follows:

Major cardiac abnormality:

- A reduction in ejection fraction to less than 50% (or LV fractional shortening of less than 28%).
- Regurgitant valvular lesion of more than mild severity.
- Valvular stenosis.
- Raised pulmonary artery pressure (>40 mmHg) or right heart dilatation.

Minor cardiac abnormalities:

- Other reported anomalies including the presence of valve leaflet thickening and regional left ventricular wall motion abnormality.

Major respiratory abnormality:

- *Restrictive defects*: Total lung capacity reduced (normal range is 80–120% predicted) with preservation of FEV_1_/FVC ratio >80%, but evidence of reduced TLCO.
- *Obstructive defect*: FEV_1_/FVC <70%.

Minor respiratory abnormality:

- Isolated reduction in TLCO (<80%).

### Statistical analysis

Descriptive statistics were calculated using SPSS v11.

## RESULTS

### Cohort characteristics

A total of 131 women previously treated with mantle radiotherapy for HL was identified, and of these, 11 were deceased, eight had a history of breast cancer and 11 were lost to follow-up. The remaining 101 were invited to attend a specialist clinic for advice about breast cancer as part of the UK ‘Patient Notification Exercise’, and 24 declined or did not respond. A total of 77 patients attended, and of these, 58 women agreed to participate in the late effects study (Table 1).

### Mortality

Of the initial cohort of 131 patients, 12 (9.2%) died including one woman with oesophageal squamous carcinoma diagnosed during the study. Five patients died from cardiac causes, four from secondary cancers, two from respiratory causes and one from infection.

### Second neoplasms

Of the 131 patients, 13 (9.9%) were identified to have had a second cancer. Eight had a history of breast cancer, two of whom died from metastatic disease, two patients had a history of basal cell carcinomas in the radiation field and one patient each had an oesophageal squamous carcinoma, cardiac sarcoma and lung cancer, respectively.

### Breast screening

Thus far, no incident breast cancers have been identified at the first breast screen.

### Late effects study

Most of the 58 patients included in the late effects study received a radiation dose of 35 Gy in 19 fractions. Of whom, 42 (72%) also received chemotherapy (10 different regimens, the most frequent being LOPP (LOPP=Chlorambucil, Vincristine, Procarbazine, Prednisolone) and MOPP (MOPP=Nitrogen Mustard, Vincristine, Procarbazine, Prednisolone)). Also, 24 women received doxorubicin and 19 women received bleomycin. as a result of relapse, seven women also received second-line chemotherapy.

#### Skin

No new cancers were detected during clinical examination, although one person was referred to dermatology with a suspicious lesion that was shown to be benign.

#### Thyroid and parathyroid

In all, 21 patients (36.2%) were already receiving thyroid replacement. Six (10.2%) were found to have a previously undiagnosed goitre or thyroid nodule and four (6.8%) untreated women were found to have a raised TSH level, suggesting undiagnosed hypothyroidism. Of those patients with goitres, all have undergone surgery with no evidence of malignancy on histology. Parathyroid hormone and calcium levels were normal in all patients.

#### Cardiovascular

In all, 10 patients (17.2%) had a major cardiac abnormality: five had evidence of mixed aortic abnormality; three had mitral regurgitation, one had a mitral/aortic valve replacement; and five had a reduction in ejection fraction of less than 50% (Table 2).

#### Respiratory function

Twenty patients had a major respiratory abnormality: fourteen women (24%) had evidence of airway obstruction and six women (10.3%) had restrictive lung defect (Table 2).

#### Incidence of medical problems

Overall, 42 (72%) of the 58 women who underwent detailed evaluation had a previously undiagnosed medical problem (Table 2).

## DISCUSSION

In a cohort of HL women, mean age 40 years, previously treated with mantle radiotherapy, 9.2% had died a nonlymphoma-related death and we found a high incidence of undiagnosed medical problems in survivors. Impressively, no patient had died of HL. At a mean of 17 years post-treatment, 72% of women had a medical problem that could relate to their previous treatment. In addition to radiotherapy, chemotherapy had also been received by 72% of patients and it should be recognised that this may contribute to some late effects (Henry-Amar, 2000). In addition, it is possible that there is a genetic risk for both HL and late effects, although to date there is no evidence to support an underlying association between the two.

We found a 9.9% cumulative incidence of secondary malignancy. This incidence is similar to the 10.6% reported at 20 years post-treatment in adult survivors of childhood HL (Bhatia *et al*, 2003), although lower than 21.9% at 25 years in a population of 32 591 survivors of HL (Dores *et al*, 2002). The most common cancer was breast cancer, as reported previously (Kenney *et al*, 2004). Increasing age over 50 years is associated with a greater cancer burden (Dores *et al*, 2002) and one of our patients aged 57 years was diagnosed and died from oesophageal cancer during the course of our study. None of this cohort developed leukaemia. The risk of leukaemia in patients treated for HL has been estimated at 0.3% after radiotherapy alone, 2.8% after chemotherapy alone and 5.4% in combined modality therapy (Brusamolino *et al*, 1998). A more recent study (Okines *et al*, 2005) estimated the incidence of second primary malignancies in patients treated for HL, including the risk of haematological malignancy. This risk was low at only 0.55% and this is consistent with our findings.

Radiation-induced damage to the heart has been recognised for some time (Gustavsson *et al*, 1990; Hancock *et al*, 1993b; Adams *et al*, 2004). The spectrum of damage ranges from constrictive pericarditis, significant valvular defects, to reduced systolic dysfunction. In the largest study (Hancock *et al*, 1993b), of 2232 Hodgkin's patients, 3.9% died from heart disease and the relative risk for heart disease was 3.1. The risk was greater with increased mediastinal radiation and the risk of myocardial infarction increased with duration of time after treatment. Our results confirm the high incidence of cardiac abnormalities in this patient group.

In the lungs, mediastinal radiation appears to create predominantly a restrictive rather than obstructive defect (Allavena *et al*, 1992; Gustavsson *et al*, 1992; Nysom *et al*, 1998). Hodgkin's patients (64–84%) treated with mantle radiotherapy had a reduction in lung perfusion and perfusion appeared to be more affected than ventilation, suggesting a primary vascular lesion (Allavena *et al*, 1992; Gustavsson *et al*, 1992). However, in patients treated during childhood, there was a reduction in lung volumes as well as transfer factor (Nysom *et al*, 1998). Our results are consistent with these observations, although we found a higher incidence of obstructive defects. This is likely to relate to confounding environmental factors, including smoking and occupation.

Hypothyroidism, thyroid nodules and thyroid cancer are well-recognised complications of mantle radiotherapy (Morgan *et al*, 1985; Sklar *et al*, 2000). Consistent with this, we found that 36.2% of Hodgkin's patients were already receiving thyroxine treatment. However, of concern, we found clinically apparent undetected thyroid nodules in 10.2% of patients and raised TSH levels in 6.8% of patients. In the data from the Childhood Cancer Survivor Study, hypothyroidism was the most common disturbance with a relative risk of 17.1; the relative risk of thyroid nodules compared to a sibling was 27 and thyroid cancer 18 (Sklar *et al*, 2000). We found no abnormality of calcium homeostasis, suggesting that the parathyroid glands are relatively resistant to radiation-induced damage, consistent with previous reports (Miltenyi *et al*, 2004).

We have screened patients treated with mantle radiotherapy as part of a national recall for breast cancer screening. In the process, we have identified that this patient group had a high incidence of undiagnosed late effects of cancer therapy. The mean age of our patients was 40 years and it is likely that with age, they will have an increasing burden of late effects and therefore the need for follow-up increases with time from the end of treatment.

## Figures and Tables

**Table 1:** Characteristics of women previously treated with mantle radiotherapy for HL who participated in late effects study

	**Detailed late effects study**		
**Characteristic**	***N***	**Mean (range)**	**s.d.**
Age at diagnosis (years)	58	23.3 (7–34)	5.8
Time since diagnosis (years)	58	17 (4–36)	8.8
Height (cm)	58	164.6 (151–180)	6.4
Weight (kg)	56	73.3 (47.7–134.0)	16.7

HL=Hodgkin's lymphoma; s.d.=standard deviation.

**Table 2:** Summary of those identified with abnormalities (patient may have more than one abnormality)

**Age at study (years)**	**Number of years of follow-up**	**Mantle radiation dose (Gy) (fractionation)**	**Cumulative dose of doxorubicin (mg m^−2^)**	**Thyroid abnormality**	**Echo abnormality**	**Lung defects**
50.1	25	35 (19)		Goitre	Major	Restrictive
30.7	12	38.5 (22)	393.0		Minor	Obstructive
34.7	17	35 (19)	240.0			Obstructive
39.1	18	35 (20)				Reduced TLCO
38.2	12	35 (20)	330.0			Restrictive
47.4	21	35 (20)			Minor	
40.9	9	35 (20)	183.0			Reduced TLCO
36.5	6	35 (20)	204.0			Reduced TLCO
47.2	14	35 (20)	360.0	Raised TSH		
37.0	5	35 (20)	240.0		Minor	
46.7	17	35 (19)	195.0			Obstructive
46.9	26	30 (19)			Minor	Obstructive
52.6	35	35 (20)			Major	Obstructive
41.4	7	35 (20)	236.0			Obstructive
41.9	9	35 (20)	284.0		Minor	Obstructive
43.2	12	35 (20)				Obstructive
36.3	9	35 (20)	195.0			Obstructive
54.2	33	35 (20)			Major	Obstructive
47.3	25	35 (20)			Minor	Obstructive
53.4	29	37.5 (24)		Raised TSH		Reduced TLCO
37.8	23	35 (19)				Restrictive
38.6	21	35 (20)		Goitre	Minor	Restrictive
36.1	16	35 (20)				Reduced TLCO
41.6	16	35 (20)	270.0		Major	
27.1	10	35 (20)	225.0			Restrictive
56.9	36	27.4 (20)			Major	Restrictive
53.6	27	35 (19)		Goitre	Major	
40.6	27	35 (18)		Goitre		
41.6	24	35 (19)		Goitre		
39.2	8	35 (20)				Reduced TLCO
47.4	23	35 (19)			Major	Obstructive
36.6	10	35 (20)				Reduced TLCO
33.4	16	35 (20)	480.0		Minor	Reduced TLCO
34.5	10	36.75 (21)			Major	Reduced TLCO
41.6	10	35 (20)	480.0	Raised TSH		Reduced TLCO
50.0	31	35 (20)		Goitre	Major	Obstructive
47.8	29	35 (20)			Minor	
42.5	24	35 (20)			Minor	Obstructive
36.5	17	35 (20)				Reduced TLCO
43.6	28	35 (19)				Reduced TLCO
35.3	9	35 (20)		Raised TSH		
47.7	19	35 (19)			Major	

TSH=thyrotropin; TLCO=transfer factor.

Major cardiac abnormalities include: aortic valve disease, mitral regurgitation, mitral and aortic valve replacement and left ventricular impairment (EF<50%).

Minor cardiac abnormalities include: valve leaflet thickening, regional abnormality of LV wall, mild aortic or mitral valvular regurgitation.

## References

[bib1] Adams MJ, Lipsitz SR, Colan SD, Tarbell NJ, Treves ST, Diller L, Greenbaum N, Mauch P, Lipshultz SE (2004) Cardiovascular status in long-term survivors of Hodgkin's disease treated with chest radiotherapy. J Clin Oncol 22: 3139–3148 15284266 10.1200/JCO.2004.09.109

[bib2] Allavena C, Conroy T, Alletti P, Bey P, Lederlin P (1992) Late cardiopulmonary toxicity after treatment for Hodgkin's disease. Br J Cancer 65: 908–912 1616862 10.1038/bjc.1992.190 PMC1977758

[bib3] Bhatia S, Robison LL, Oberlin O, Greenberg M, Bunin G, Fossati-Bellani F, Meadows AT (1996) Breast cancer and other second neoplasms after childhood Hodgkin's disease. N Engl J Med 1996: 745–751 10.1056/NEJM199603213341201 8592547

[bib4] Bhatia S, Yasui Y, Robison LL, Birch JM, Bogue MK, Diller L, DeLaat C, Fossati-Bellani F, Morgan E, Oberlin O, Reaman G, Ruymann FB, Tersak J, Meadows AT (2003) High risk of subsequent neoplasms continues with extended follow-up of childhood Hodgkin's disease: report from the Late Effects Study Group. J Clin Oncol 21: 4386–4394 14645429 10.1200/JCO.2003.11.059

[bib5] Brusamolino E, Anselmo AP, Klersy C, Santoro M, Orlandi E, Pagnucco G, Lunghi F, Maurizi-Enrici R, Baroni CD, Lazzarino M, Mandelli F, Bernasconi C (1998) The risk of acute leukemia in patients treated for Hodgkin's disease is significantly higher after combined modality programs than after chemotherapy alone and is correlated with the extent of radiotherapy and type and duration of chemotherapy: a case-control study. Haematologica 83: 812–823 9825578

[bib6] Deniz K, O'Mahony S, Ross G, Purushotham A (2003) Breast cancer in women after treatment for Hodgkin's disease. Lancet Oncol 4: 207–214 12681264 10.1016/s1470-2045(03)01033-7

[bib7] Dores GM, Metayer C, Curtis RE, Lynch CF, Clarke EA, Glimelius B, Storm H, Pukkala E, van Leeuwen FE, Holowaty EJ, Andersson M, Wiklund T, Joensuu T, van't Veer MB, Stovall M, Gospodarowicz M, Travis LB (2002) Second malignant neoplasms among long-term survivors of Hodgkin's disease: a population-based evaluation over 25 years. J Clin Oncol 20: 3484–3494 12177110 10.1200/JCO.2002.09.038

[bib8] Gustavsson A, Eskilsson J, Landberg T, Larusdottir H, Svahn-Tapper G, White T, Wollmer P (1992) Long-term effects on pulmonary function of mantle radiotherapy in patients with Hodgkin's disease. Ann Oncol 3: 455–461 1498064 10.1093/oxfordjournals.annonc.a058234

[bib9] Gustavsson A, Eskilsson J, Landberg T, Svahn-Tapper G, White T, Wollmer P, Akerman M (1990) Late cardiac effects after mantle radiotherapy in patients with Hodgkin's disease. Ann Oncol 1: 355–363 2261376 10.1093/oxfordjournals.annonc.a057774

[bib10] Hancock SL, Tucker MA, Hoppe RT (1993a) Breast cancer after treatment of Hodgkin's disease. J Natl Cancer Inst 85: 25–31 8416252 10.1093/jnci/85.1.25

[bib11] Hancock SL, Tucker MA, Hoppe RT (1993b) Factors affecting late mortality from heart disease after treatment of Hodgkin's disease. J Am Med Assoc 270: 1949–1955 8411552

[bib12] Henry-Amar M (2000) Long-term problems. In Malignant Lymphoma, Hancock BW, Selby PJ, MacLennan K, Armitage JO (eds), Vol. 1, pp 421–436. London: Arnold

[bib13] Henry-Amar M, Joly F (1996) Late complications after Hodgkin's disease. Ann Oncol 7(Suppl 4): 115–126 10.1093/annonc/7.suppl_4.s115 8836422

[bib14] Horwich A, Swerdlow AJ (2004) Second primary breast cancer after Hodgkin's disease. Br J Cancer 90: 294–298 14735166 10.1038/sj.bjc.6601499 PMC2409557

[bib15] Kenney LB, Yasui Y, Inskip PD, Hammond S, Neglia JP, Mertens AC, Meadows AT, Friedman D, Robison LL, Diller L (2004) Breast cancer after childhood cancer: a report from the Childhood Cancer Survivor Study. Ann Int Med 141: 590–597 15492338 10.7326/0003-4819-141-8-200410190-00006

[bib16] Miltenyi Z, Keresztes K, Lakos G, Varoczy L, Miltenyi L, Illes A (2004) Is the treatment of Hodgkin's disease detrimental to the parathyroid gland? Acta Haematol 112: 148–151 15345897 10.1159/000079726

[bib17] Morgan GW, Freeman AP, McLean RG, Jarvie BH, Giles RW (1985) Late cardiac, thyroid and pulmonary sequelae of mantle radiotherapy for Hodgkin's disease. Int J Radiat Oncol Biol Phys 11: 1925–1931 3932270 10.1016/0360-3016(85)90273-1

[bib18] Nysom K, Holm K, Hertz H, Hesse B (1998) Risk factors for reduced pulmonary function after malignant lymphoma in childhood. Med Pediatr Oncol 30: 240–248 9473759 10.1002/(sici)1096-911x(199804)30:4<240::aid-mpo6>3.0.co;2-g

[bib19] Okines A, Thomson CS, Radstone CR, Horsman JM, Hancock BW (2005) Second primary malignancies after treatment for malignant lymphoma. Br J Cancer 93: 418–424 16106249 10.1038/sj.bjc.6602731 PMC2361580

[bib20] Sklar C, Whitton J, Mertens A, Stovall M, Green D, Marina N, Greffe B, Wolden S, Robison L (2000) Abnormalities of the thyroid in survivors of Hodgkin's disease: data from the Childhood Cancer Survivor Study. J Clin Endocrinol Metab 85: 3227–3232 10999813 10.1210/jcem.85.9.6808

[bib21] Swerdlow AJ, Barber JA, Hudson GV, Cunningham D, Gupta RK, Hancock BW, Horwich A, Lister TA, Linch DC (2000) Risk of second malignancy after Hodgkin's disease in a collaborative British cohort: the relation to age at treatment. J Clin Oncol 18: 498–509 10653865 10.1200/JCO.2000.18.3.498

[bib22] Travis LB, Hill DA, Dores GM, Gospodarowicz M, van Leeuwen FE, Holowaty E, Glimelius B, Andersson M, Wiklund T, Lynch CF, Van't Veer MB, Glimelius I, Storm H, Pukkala E, Stovall M, Curtis R, Boice Jr JD, Gilbert E (2003) Breast cancer following radiotherapy and chemotherapy among young women with Hodgkin disease. J Am Med Assoc 290: 465–475 10.1001/jama.290.4.465 12876089

[bib23] Wolden SL, Lamborn KR, Cleary SF, Tate JT, Donaldson SS (1998) Second cancers following pediatric Hodgkin's disease. J Clin Oncol 16: 536–544 9469338 10.1200/JCO.1998.16.2.536

